# Winter weight loss of different subspecies of honey bee *Apis mellifera* colonies (Linnaeus, 1758) in southwestern Sweden

**DOI:** 10.1371/journal.pone.0258398

**Published:** 2021-10-14

**Authors:** Niclas Norrström, Mats Niklasson, Sonja Leidenberger

**Affiliations:** 1 School of Bioscience, Department of Biology and Bioinformatics, University of Skövde, Skövde, Sweden; 2 Stiftelsen Nordens Ark, Åby säteri, Hunnebostrand, Sweden; King Khalid University, SAUDI ARABIA

## Abstract

Honey bees are currently facing mounting pressures that have resulted in population declines in many parts of the world. In northern climates winter is a bottleneck for honey bees and a thorough understanding of the colonies’ ability to withstand the winter is needed in order to protect the bees from further decline. In this study the influence of weather variables on colony weight loss was studied over one winter (2019–2020) in two apiaries (32 colonies in total) in southwestern Sweden with weather stations recording wind and temperature at 5-min intervals. Three subspecies of honey bees and one hybrid were studied: the native *Apis mellifera mellifera*, the Italian *A*. *m*. *ligustica*, the Carniolan *A*. *m*. *carnica* and the hybrid Buckfast. Additionally, we recorded *Varroa* mite infestation. To analyze factors involved in resource consumption, three modelling approaches using weather and weight data were developed: the first links daily consumption rates with environmental variables, the second modelled the cumulative weight change over time, and the third estimated weight change over time taking light intensity and temperature into account. Weight losses were in general low (0.039 ± 0.013kg/day and colony) and comparable to southern locations, likely due to an exceptionally warm winter (average temperature 3.5°C). Weight losses differed only marginally between subspecies with indications that *A*. *m*. *mellifera* was having a more conservative resource consumption, but more studies are needed to confirm this. We did not find any effect of *Varroa* mite numbers on weight loss. Increased light intensity and temperature both triggered the resource consumption in honey bees. The temperature effect on resource consumption is in accordance with the metabolic theory of ecology. The consequences of these findings on honey bee survival under predicted climate changes, is still an open question that needs further analysis.

## Introduction

The European honey bee (*Apis mellifera* L.) has developed unique adaptation strategies to cope with cold climates. These adaptations include: modification of colony cycle, maximizing its own reproductive success, long-term resource storage and thermoregulation [1]. The bee colony is using both behavioral and physiological responses for its thermoregulation, through an increase in metabolic heat production and a clustering behavior [2]. Each individual of the cluster is contributing to the superorganism’s thermoregulation through microvibration by its flight muscles of the thorax [3]. The advantage of a superorganism is that workers by themselves can carry on collective functions directly and therefore react faster to stress (e.g. temperature changes) [4, 5]. When it comes to energy balance it has been shown that the body mass scaling of the metabolic rate is similar for individuals and whole colonies of social insect species [6].

The bees’ ability to survive the winter depends on a combination of creating an insulating layer of bees at the edge of the winter cluster, endothermic heat production by bees, primarily in the central parts of the cluster, and on the ectothermic bees’ resting metabolism [5]. The winter in northern climates pose serious challenges and is a major bottleneck for colony survival. The bees need to collect enough honey to ingest throughout the winter in order to produce heat that keeps the queen warm and alive. Other important factors for surviving winter are for example beehive properties (e.g. insulation and ventilation), mites (like *Varroa destructor*), pathogens (fungi, bacteria and viruses) and preceding summer weather conditions [7–10]. Beside temperature, several studies have emphasized the importance of light intensity for controlling the life cycle in honey bees e.g. [1, 11]. After the midwinter, when temperature typically is at its lowest in the Northern Hemisphere (January-February), honey bees can have a mid-winter brood. A study observed that the colonies which utilize this strategy tend to have larger populations and early swarming [12]. The timing of the brood onset in late winter is a critical step for bee colonies where a well-timed brood will result in larger honey storages [13].

Thermoregulation energy expenditure is usually approximated with beehive weight loss, typically recorded by beehive scales [12, 14–16]. Different studies have shown that honey bees can lose up to 26 kg in colder climates [1] and around 12 kg in warmer regions [16]. The metabolic rate of a colony has been shown to be affected by ambient temperature and other biotic factors (e.g. brood and clustering) [1, 17]. Several attempts have been done to model the weight loss of bee colonies, where energy expenditure model approaches have been developed to describe the self-organized thermoregulation of the individual bees in the winter cluster by taking local bee density, temperature and individual mortality into account, e.g. [18, 19]. However, these models are qualitative approaches not based on empirical data. When it comes to quantitative models, ecological processes often show non-linear relationships which are important to consider. Such ecological systems are well suited to use non-linear mixed effects models, but this can easily lead to high complexity when interpreting the results, e.g. [20–22].

Given the many reports on widespread colony collapse and winter losses in recent years [8, 23, 24], there is an urgent need to explore the role of local adaptation in honey bees [25–27]. Not many studies have observed the winter challenges in colder climates of many colonies and different subspecies of *Apis mellifera* simultaneously. In this study, we aimed to investigate weight dynamics in three *A*. *m*. subspecies and one hybrid over one winter (October 2019—March 2020) in two Swedish apiaries and analyzed its relation to environmental variables (temperature, light intensity, wind and colony size) as well as *Varroa* mite infestation. We developed three different modelling approaches in order to gain a better understanding of resource consumption in honey bee winter clusters.

## Material and methods

### Study sites, apiaries and bee colonies

In 2019, two apiaries with 16 beehives in each were established in two different localities in southwestern Sweden, one at Uddevalla (Lat/Long 58.295922°N and 11.992339°E, 76 m asl) and one at Nordens Ark (Lat/Long 58.442481°N and 11.437202°E, 25 m asl). The landscapes are very similar at the two apiaries with small difference in climate, flora (meadows, deciduous and coniferous forests) and geological substrates. The apiary at Nordens Ark has a slightly more oceanic climate than the Uddevalla apiary.

**Fig 1:**
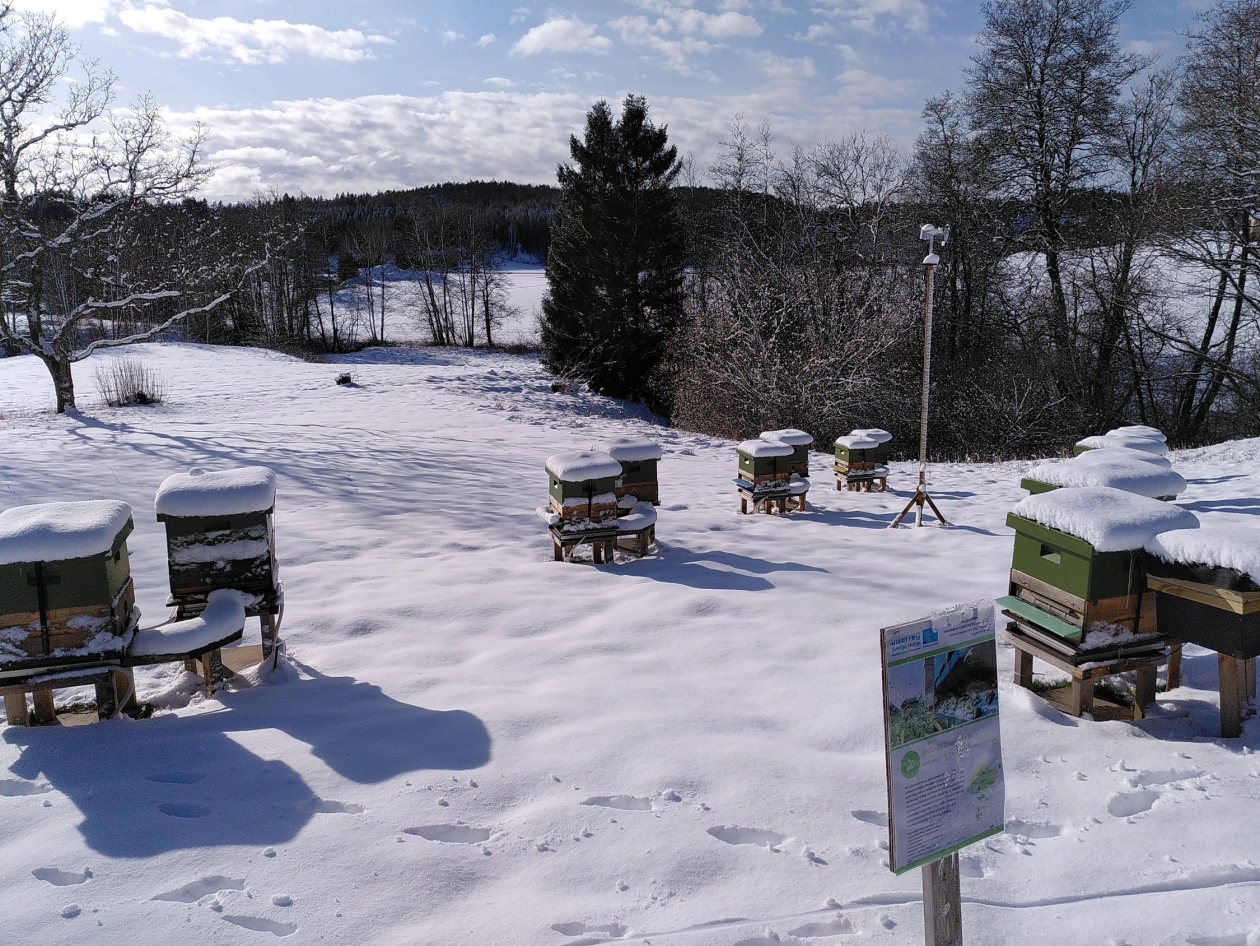
Arrangement of the 16 beehives in two shifted lines in the apiary. The weather station is placed in the middle. The picture of the apiary at Uddevalla was taken on the 26^th^ of February 2020.

When setting up the experiment (summer 2019), the colonies started with the same number of worker bees (around 10 000 individuals plus 5–6 brood frames) and one honey frame for each colony. The beehives were placed in two shifted lines, where each line was formed by eight beehives (Fig 1). Two beehives each were put on wooden racks 35cm above the grass covered ground. The beehives were installed with their entrances at an angle of approximately 90° away from each other with the entrances of the beehives orientated to the southwest and to the southeast, respectively. The distances between the middle of the entrances of the beehives measured 1.5m. The distance between each wooden rack was 3m, and between the two lines 4m (Fig 1).

The colonies were formed between June-August 2019. Sister queens of each subspecies and the hybrid were equally shared between both apiaries in order to control the genetic variability. Three subspecies of *Apis mellifera*, namely *A*. *m*. *carnica* (= *CAR*), *A*. *m*. *ligustica* (= *LIG*), *A*.*m*. *mellifera* (= *MEL*) and the hybrid Buckfast (= *BUCK*) were used in the experiment. The selection of queen material was performed with the help of the national beekeepers’ association for each subspecies and *BUCK*. The different associations are following the Swedish bee breeding standards (e.g. for wing indices and morphology of the bee, calibration tests on cake strength, harvest, swarm inertia, temperament and documentation of *Varroa* infestation). To keep genetic variability for each subspecies as high as possible, queen material from three different national queen breeders and one international queen breeder were chosen to represent each subspecies in the experiment. Voucher specimens are deposited in the Hymenoptera collection of the Department of Zoology, Swedish Museum of Natural History (SMNH), Stockholm, Sweden (Nos. NHRS-HEVA000017564-17579 Uddevalla, NHRS-HEVA000017580-17611 NordensArk).

### Beehives, measurement techniques and time interval

Each bee colony was housed in a so called Swedish Lågnormal (LN) beehive body (outer measures 462x462mm, inside measures 382x382x230mm) with an isolated roof. Each box housed 10 frames with the Swedish LN standard sizes of 366x222mm. The beeswax sheets followed the LN standard of 342x197mm and cell size 5.1 mm. Both the beehive body and the roof were of polystyrene. The bottom of the beehive was put on a pollen catcher (sizes: 470x470x215mm) of wood, inactivated during winter season. To minimize the number of error flights, the beehive entrances were painted with different colors (yellow, green, blue and lilac). Each subspecies replicate (4 in total) was situated in a beehive with a different colored entrance. The colonies were distributed in such a way that they had the same numbers of inside and outside positions in the two lines of the apiary to minimize possible edge effects. After the honey harvest (12^th^ of September, about 1–3 frames only), the colonies were fed with 55%-60% sugar solution (water: sugar = 2:3) over one week *ad libitum* (around 12–15 kg). In the autumn, all beehives were positioned on a scale system (model ApiGraph 3.1, Wolf-Waagen GmbH & Co, Germany), that measured the total weight in a 5-minute interval and recorded with an accuracy of 0.01kg. Additionally, in each apiary a 5-in-1 weather station (model ApiWeather-RF6, Wolf-Waage GmbH & Co, Germany) were installed at 3m height, which at 5-minute intervals recorded the local temperature (°C) and wind direction.

To survey the intensity of *Varroa* mite infestation (*V*. *destructor*, Anderson & Trueman, 2000) per colony, an oxalic acid treatment was performed in late autumn (29^th^ of November), when the colonies were definitively without brood. The oxalic acid (3.2% solution) was dropped between the frames (20-35ml/colony, depending on the colony size) directly on the individuals forming the winter cluster. At the same time, the size class of the colony was noted (A: a small colony, covering less than 1/3 of the wax frames; B: a medium colony covering around the half of wax frames and C: a large colony covering more than the half of the frames—when viewed from above). One colony was observed to be without a queen in autumn (E: special case). A yellow *Varroa* grate was placed at the bottom of each beehive and after 10 days the grates were visually counted for dropped and dead mites.

The analyzed time frame in the experiment was in total 20 weeks (140 days), from the 26^th^ of October 2019 until the 15^th^ of March 2020.

### Data series on light intensity and temperature

The output from the Swedish STRÅNG-model was used as a measure of light intensity, namely photo active radiation (PAR) (W/m^2^), in the analyses. The STRÅNG-output is provided by the Swedish Meteorological and Hydrological Institute. It is a mesoscale model for solar radiation with a resolution of 2.5 x 2.5 km from March 2017 onwards (for more information about the model see http://strang.smhi.se/). The time series were extracted in a daily time resolution for each locality.

**Fig 2:**
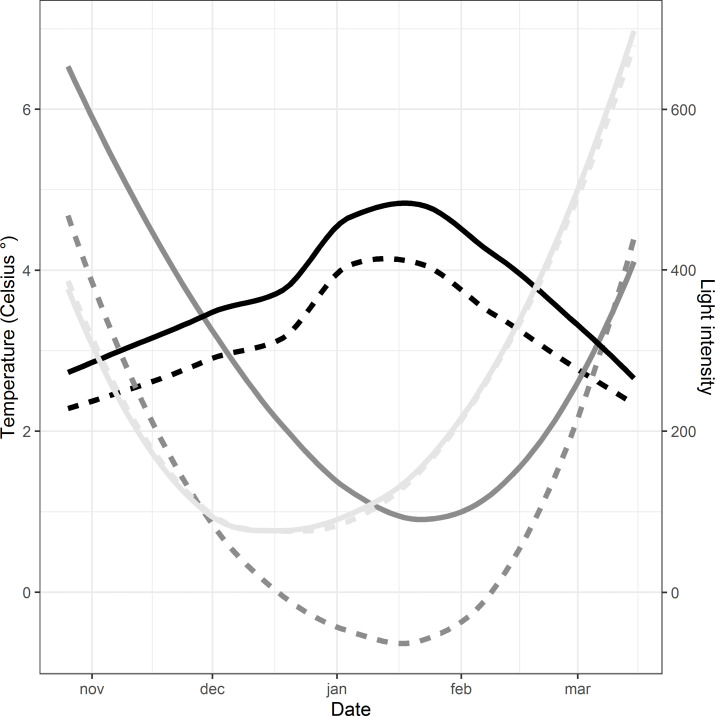
Measured air temperature (°C) (black) and reference temperatures (°C) (dark grey) at Uddevalla (dotted) and Nordens Ark (solid) over the total experiment of 20 weeks (5 months), from the 26^th^ of October 2019 until the 15^th^ of March 2020. Additionally, light intensity, namely photo active radiation ((PAR) (W/m^2^), light grey) at both locations is presented on the second y-axis. The reference temperature lines are based on the monthly average temperatures at weather station “Måseskär A” (data measured between August 1995 and December 2020) for Nordens Ark and “Åsbräcka-Torpabron V” (measured between August 1997 and December 2015) for Uddevalla and the data has been fitted to polynomial functions. Temperature and light intensity lines are shown as running averages to present the general trends in the data.

Additionally to the measured temperature data by the experiment’s own weather station at each bee yard, long time temperature data series were downloaded from the Swedish Meteorological and Hydrological Institute (SMHI) of the nearest weather station (Uddevalla: Åsbräcka-Torpabron V and Nordens Ark:Måseskär A) (from August 1997—December 2015 and August 1995- December 2020, respectively) (Fig 2).

### Data manipulation

The STRÅNG model was missing data points for the 31^st^ of December 2019 for both localities due to the turn of the year. Following the advice of the STRÅNG team (pers. comm. T. Carlund, SMHI) we used the same PAR intensity value as for the 4^th^ of January instead, because the weather situation was relatively similar for both days in the analyzed region.

At the locality Nordens Ark data loss occurred for the main scale system on the 4^th^ of March 2020 due to power/technical failure. The missing weight data were replaced with the value from the day before.

Between the 25^th^ of February and the 3^rd^ of March, there was snow on the beehives in the Uddevalla apiary (Fig 1), which increased the weight on all beehives considerably. To resolve this problem, the weight of each beehive was adjusted to remain constant at the weight on 25/2 until 3/3. The corresponding yield values were set to 0. On other occasions slight positive weight gains due to environmental variations were recorded but these were considered variations in the data and were not corrected.

### Statistical analysis

All statistical analyses and models were run in R v. 4.0.5 [28]. The packages and functions will be named under each analysis. We analyzed the amount of variance by running several factorial ANOVA [29]. In cases of unbalanced design, we preferred the approach Type II sums of squares (SS) over Type III SS following the recommendation by [30]. First, the weight loss during the entire experiment time was log-transformed to stabilize variances [31] and then tested for effects of subspecies, class and locality. Secondly, the number of *Varroa* mites (counts of numbers of individuals) was sqrt-transformed to stabilize variances [31] and tested for effects of subspecies, class and locality. Both designs were unbalanced (ANOVA, type II SS) and the car package (v. 3.0–10) was used (ANOVA function). The subsets of subspecies (*BUCK*, *CAR*, *LIG*, *MEL*) for the factor *class* were post-analyzed by a simple ANOVA in both runs.

### Model descriptions

To analyze the factors influencing the weight loss during winter in the four subspecies, we developed three different modelling approaches: a) *daily consumption models* (DCMs), b) *total weight models* (TWMs) and c) *weight assessment models* (WAMs).

#### a) Daily consumption models (DCMs)

Non-linear, linear and multiple linear DCMs were developed and evaluated against each other.

Each winter cluster was treated as a superorganism instead of considering each individual bee. In a winter cluster the bees are assumed to not spend any relevant amount of energy on somatic growth or reproduction (e g. the growth of reproductive organs). In our analysis energy was assumed to be used exclusively for the maintenance of bees and heat production. The individual metabolic requirement of such an organism (*R*) can be described by the master equation of metabolic theory of ecology (MTE) [32]:

$$R=aW^{b}e^{\frac{-\varepsilon }{\kappa T}}$$ (Eq 1)

where the metabolic activity expenditure rate *aW*^*b*^ is assumed to relate to body mass (W) according to a power law. Parameter *a* is a normalization constant and *b* is the allometric exponent. Both *a* and *b* can vary and the allometric constant *b* typically lies within the interval 1/2 ≤ b 1 [33] where *b* = 3/4 is commonly applied [32]. The Boltzmann-Arrhenius factor e^-ε/(κT)^ accounts for the increase in chemical reaction rates as absolute temperature *T* increases where *ε* is the activation energy and *κ* is Boltzmann’s constant [34, 35].

The beehive provides some isolation but the temperature right next to the winter cluster is typically only marginally above the ambient temperature [5], which is why we modelled the utilization of food for heating without the complexities of the insulation effects from the beehive. A honey bee cluster also isolates itself effectively with bees at the surface of the cluster acting as insulators where larger clusters of bees have better insulation [36].

Since precise data about the weight of each winter cluster (*i*) was not available, the weight based metabolic activity expenditure rate in our models (*W*_*i*_) was considered constant, where each cluster has an individual metabolic activity expenditure rate. The individual metabolic requirement used by the clusters in the models were:

$$R_{i}=W_{i}e^{\frac{-\varepsilon }{\kappa T}}$$ (Eq 2)

Simplifying the metabolic activity expenditure rate removes all possible interactions between *a* and *b*, where e g. the metabolic-level boundary hypothesis has theorized that the metabolic exponent *b* varies with activity level *a* [37]. Since our data did not contain direct information about the activity level, these dynamic interactions were not considered here, however winter clusters appear to experience daily cycles of activity [17] and a varied temperature in the clusters suggests varying activity levels [38, 39].

The change in weight between two consecutive days was termed yield (*Y*), where a positive yield corresponds to an increase in weight and consequently a negative value represents loss of weight. Negative yield in our models thus represents the number of resources utilized by the bees between two consecutive days.

The temperature based metabolic activity expenditure rate (*W*_*i*_, Eq 2) was used to predict the yield from each beehive where *W*_*i*_ is optimized. Since yield is negative when honey or sugar water is consumed, the yield equation that we fitted our data to was:

$$-Y_{i}=W_{i}e^{\frac{-\varepsilon }{\kappa T}}$$ (Eq 3)

The value of ε is expected to lie in the interval 0.6 < ε < 0.7 according to [40], where the mean value ε = 0.65 is often applied and we used it in this study. The value of the Boltzmann’s constant κ is 8.617∙10^−5^ eV/K [34]. The temperature values used are the average temperatures (in Kelvin) of the locations’ climate stations. To account for the individual weights and activation patterns of the different colonies the statistical model was a non-linear mixed-effects model with the identity of the colonies as a random effect. The residual errors were assumed to be Gaussian random variables with mean 0 and they were assumed to be independent and identically distributed. The residuals showed no clear trend over temperature so no autocorrelation structure was evaluated.

We also investigated if simplifying the model to a linear regression model over temperature could fit the data equally well. Furthermore, we evaluated multiple linear regression models of yield including environmental parameters. By evaluating all additive combinations of the variables temperature (T), light intensity (L), wind direction (W) and colony size class (C), including all their pairwise and three-way interactions 32 768 multiple linear regression models were evaluated. In these models no variable was treated as a random effect, but by including colony size class as a categorical variable the effects of different winter cluster sizes could be accounted for. The temperature values used in all DCMs are the maximum temperatures from each day.

#### b) Total weight models (TWMs)

Insights about winter cluster strategies can be gained by analyzing the changes in weight over time. Due to the variable and non-linear temperature changes over the period it is difficult to integrate the temperature in the metabolic function in this case. The average temperature increases in the middle of the experiment with lower temperatures in the beginning and at the end (Fig 2). According to Eq 1 the loss of weight should thus be highest in the middle of the experiment with lower weight loss rates in the beginning and at the end. To account for this sigmoidal shape of the weight change, the following four-parameter logistic function, based on [41], was used to fit the weight data over time:

$$W_{Hive}=\frac{A-v}{1+e^{(m-t)/s}}+v$$ (Eq 4)

Where *W*_*Hive*_ is the weight of the beehive and *t* is time, measured in days. Parameters *A* and *v* are the respective lower and upper horizontal asymptotes, *m* represents the value (time) when *W*_*Hive*_ is midway between *A* and *v*, and *s* is the steepness of the function [41]. Each individual beehive has its own condition so individual beehives are treated as a grouping factor and all parameters have both fixed and random effects. The simulations utilized the R-package *nlme*. Since autocorrelation can be an issue when data are analyzed over time an autocorrelation structure of order 1 (function *corAR1*) with 0.3 as the value of the lag 1 correlation was evaluated. A simulation that included the same autocorrelation and a variance structure that allows different variances for each grouping factor (function *varIdent*) was also evaluated.

#### c) Weight assessment models (WAMs)

Another approach used to model the resource consumption was to model the development of the weight in each colony with a constant daily consumption rate to predict daily metabolism (weight loss) one day forward in time. This requires a parameter for the initial weight for each beehive from which the consumption during the first day was removed. Further time-steps reduce the weight from the previous day’s weight. By repeating this over all days the development of weight over time can be modelled. The environmental variables temperature (°C) and light (PAR W/m2) were also included by including parameters that reduce weight proportional to the environmental variable, and these parameters were both combined additively and in interaction with each other. The weight update equations used in this approach were therefore variants of the full update equation:

$$W_{Hive,t+1}=W_{Hive,t}-C_{h}-s\cdot T_{p,t}-l\cdot L_{p,t}-u\cdot T_{p,t}\cdot L_{p,t}$$ (Eq 5)

where *W*_*Hive*,*t+1*_ is the weight of a beehive at time t+1 and *C*_*h*_ is the beehive-specific, constant, daily, consumption rate, *T*_*p*,*t*_ is the location-specific average temperature at day *t*, and *L*_*p*,*t*_ is the location-specific light intensity at day *t*. Each beehive’s weight for each day is predicted with the variations of the function above (see Table 1 for the specific equations used) and the beehive-specific parameters *s*, *l* and *u* are then optimized to minimize the residual sums of square error (RSS) between the weight data and the predicted weights. The optimization used A general-purpose optimization (function *optim*) with optimization method BFGS which is a quasi-Newton method was used for optimization [28].

**Table 1:** The different models’ fit to the weight data, arranged by model type.

Type	Modelling	Model version	AIC	Log-Likelihood	df
DCM	daily yield	non-linear	239	-134	-
DCM	daily yield	linear T	200	-133	3
DCM	daily yield	linear T + L	154	-73	4
DCM	daily yield	best multiple linear	**-196**	134	36
TWM	weight loss	plain	2827	-1398	15
TWM	weight loss	corAR1	-917	474	16
TWM	weight loss	corAR1 & varIdent	**-1117**	606	47
WAM	weight loss	C_h_	-3142	-	65
WAM	weight loss	C_h_ + T_p,t_	-8909	-	97
WAM	weight loss	C_h_ + L_p,t_	-9280	-	97
WAM	weight loss	C_h_ + T_p,t_ + L_p,t_	**-9439**	-	129
WAM	weight loss	C_h_ + T_p,t_ * L_p,t_	-9303	-	97

Akaike information criterion (AIC) was used to rate the models. The AIC value of the model of each model type with the lowest AIC-value is shown in bold. Log likelihood is presented for the daily consumption model (DCM) and total weight model (TWM) where the log likelihood of the TWM models represents restricted likelihoods due to the optimization algorithm used. Degrees of freedom (df) is not shown for the non-linear DCM since degrees of freedom in models with random effects doesn’t mean the same thing as in the other models where the star specifies what degrees of freedom the algorithm considered. The AIC of the weight assessment model (WAM) is derived from residual sums of square error (RSSs) and cannot be compared to the other models’ AIC. The DCM and TWM do not have the same underlying data so AIC should not be compared between them either.

All models except the WAMs were evaluated with the likelihood based Akaike information criteria (AIC) [42, 43]. For the WAMs AIC was calculated from the RSS using the function: $AIC=\log\;\log\left(\frac{RSS}{n}\right)+2p$, where *n* is the number of samples and *p* is the number of parameters. The likelihood-based AIC values were not compared to the RSS based AIC values.

The weight of the beehives in the study was the combined weight of the material of the beehive including top lid, the honey, pollen, wax and sugar water and the weight of the bees. Weight loss of the beehives over time was considered to be mainly caused by the consumption of resources since the other factors are mainly constant. Since no substantial new resources were collected by the bees during the experiment, the weight of the beehives should constantly decrease over time. However, since some rain and snow fell on the beehives the weight increased at certain points during the time of the experiment. Also, a higher humidity can potentially have increased the weight somewhat since minor parts of the beehive construction was made of wood (e.g. the bottom and the scale plates), and a higher humidity can increase the density of the wood through the accumulation of water. The reduction of weight due to bee die off and the following evaporation of water from the dead bees has not been considered here but was assumed to be negligible since the dead bees remained in the beehive.

## Results

### Colony size distribution (class)

The colony size differed between the subspecies and locations (Table 2). Ten colonies were assessed to size class A, 11 to class B, 10 to class C and one colony was assessed to size class E (n = 32). Medium sized colonies were more common for *BUCK* size colonies (class A: 1, B: 5, C: 2, n = 8), while *CAR* (A:3, B: 3, C: 2, n = 8) and *MEL* (A:4, B:2, C: 2, n = 8) had more of small colonies. *LIG* had the highest number of large colonies (A:2, B:1, C:4) and the queenless colony (E: 1), which survived the winter (n = 8) (Table 2).

**Table 2:** The weight loss (kg) of honey bees at the two localities.

Weight loss (kg) (mean±SD)	Nordens Ark				Uddevalla				Daily weight loss (kg/day) per subspecies (mean±SD)
	A	B	C	E	A	B	C	E	
*BUCK*	NA (n = 0)	6.01±1.30 (n = 3)	5.36±0 (n = 1)	NA (n = 0)	3.77±0 (n = 1)	5.51±0.76 (n = 2)	4.67±0 (n = 1)	NA (n = 0)	0.0383±0.008 (n = 8)
*CAR*	4.46±0.75 (n = 2)	5.19±0 (n = 1)	7.34±0 (n = 1)	NA (n = 0)	3.86±0 (n = 1)	4.11±0.37 (n = 2)	8.64±0 (n = 1)	NA (n = 0)	0.0376±0.013 (n = 8)
*LIG*	5.04±0 (n = 1)	NA (n = 0)	6.56±1.07 (n = 2)	12.01±0 (n = 1)	4.13±0 (n = 1)	4.35±0 (n = 1)	6.13±1.03 (n = 2)	NA (n = 0)	0.0454±0.018 (n = 8)
*MEL*	4.50±0.19 (n = 2)	5.75±0 (n = 1)	5.22±0 (n = 1)	NA (n = 0)	3.96±0.90 (n = 2)	3.68±0 (n = 1)	7.73±0 (n = 1)	NA (n = 0)	0.0351±0.010 (n = 8)
**Daily weight loss (kg/day)** per locality (mean±SD)	0.042±0.014				0.036±0.011				

Weight loss in mean±SD for each subspecies (Buckfast hybrid bee of *Apis mellifera* (= *BUCK*), *A*. *m*. *carnica* (= *CAR*), *A*. *m*. *ligustica* (= *LIG*), *A*. *m*. *mellifera* (= *MEL*)) and size class of the colonies (small (= A), medium (= B), large (= C), special case without queen (= E)) of *Apis mellifera* at the two different localities.

### Changes in colony weight (total weight loss and daily weight loss rate)

**Fig 3:**
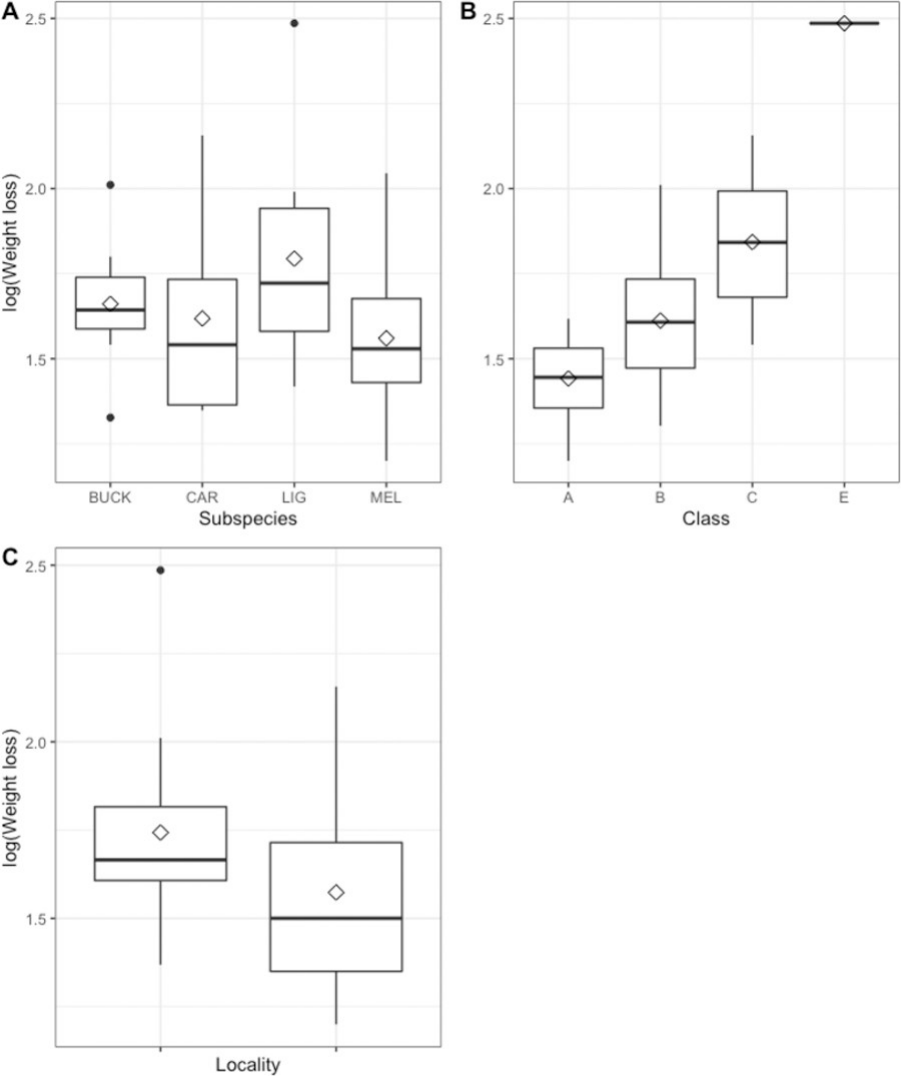
The weight loss log-transformed. Results for A) the four different subspecies (Buckfast hybrid bee of *Apis mellifera* (= *BUCK*), *A*. *m*. *carnica* (= *CAR*), *A*. *m*. *ligustica* (= *LIG*), *A*. *m*. *mellifera* (= *MEL*), B) at the different size classes of the colonies (small (= A), medium (= B), large (= C), special case without queen (= E)) and C) at the two different bee yards (in NordensArk and Uddevalla, Sweden). Median values are presented with a horizontal black bar and the interquartile range is shown as a boxplot with the range of the data as dashed lines and outliers as dots. The mean is represented by a diamond.

The average daily weight loss for all colonies in the experiment was 0.039±0.013kg/day (n = 32). In total, all colonies lost less than 9 kg each during the experiment at both localities (Fig 3 and Table 2). The only exception was the Queen-less *LIG* colony which lost a total of 12.01 kg. Weight loss ranges were for *BUCK* 3.77–7.31, for *CAR* 3.86–8.64, for *LIG* 4.13–7.63 and for *MEL* 3.68–7.73 kg, being similar at both localities. The highest daily weight loss could be found for *LIG* (0.0454±0.018 kg/day) followed by *BUCK* (0.0383±0.008), *CAR* (0.0376±0.013) and *MEL* (0.0351±0.010). At Nordens Ark the weight loss was slightly higher than at Uddevalla (Fig 3) and the daily weight loss was 0.042±0.014 *vs*. 0.036±0.011kg/day, respectively.

We could also observe that smaller colonies did not experience as much weight loss as larger colonies (Fig 3), where the effect of size class was significant (*F*_*3*,*9*_ = 15.236, *p*<0.005). In the post-analysis 1-way ANOVA, *LIG* (*F*_3,9_ = 8.815, *p*<0.005) and *CAR* (*F*_2,9_ = 9.936, *p* = 0.005) showed significance for the factor Class. The *LIG* colony, without a queen (class E), was a unique exception, but in addition *LIG* and *CAR* showed higher weight loss in larger colonies (class C) in contrast to *MEL* and *BUCK* colonies (in the same size class) (Fig 3). The other factors (Subspecies and Locality) showed no effects (p>0.05) (Fig 3 and S1 Table). In total, nine out of 32 effects were not estimable, due to missing observation (NA, Table 2).

### Varroa infestation

**Fig 4:**
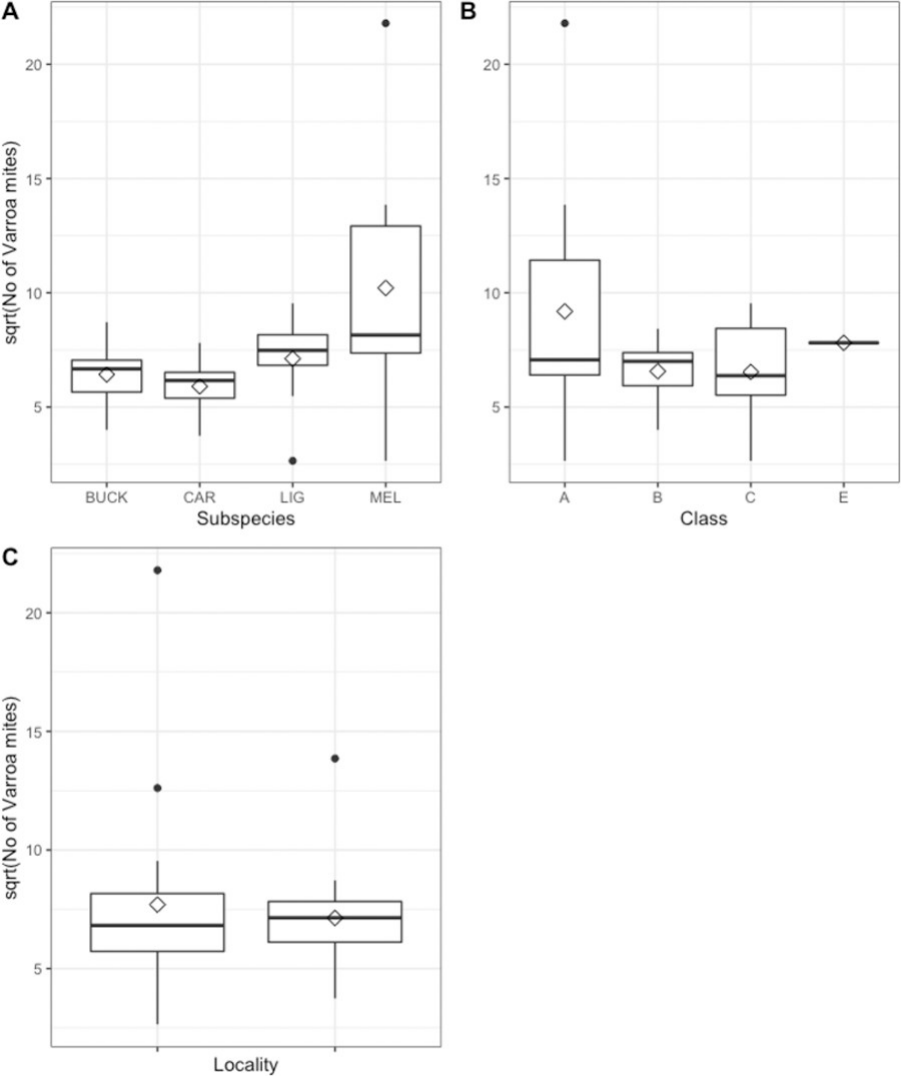
The number of *Varroa* mites sqrt-transformed. Results for A) the four different subspecies (Buckfast hybrid bee of ***Apis mellifera* (= *BUCK*)**, *A*. *m*. *carnica* **(= *CAR*)**, *A*. *m*. *ligustica* **(= *LIG*)**, *A*. *m*. *mellifera* **(= *MEL*)**, B) at the different size classes of the colonies **(small (= A), medium (= B), large (= C), special case without queen (= E))** and C) at the two different bee yards (in NordensArk and Uddevalla, Sweden). Median values are presented with a horizontal black bar and the interquartile range is shown as a boxplot with the range of the data as dashed lines and outliers as dots. The mean is represented by a diamond.

In general, the number of counted *Varroa* mites was low in the colonies, classes and subspecies with less than 100 individuals for most cases (Fig 4). The number of counted *Varroa* mites was much higher for two smaller colonies (class A) of *MEL*, where the queen originally came from a Swedish *Varroa*-free area (Umeå, Northern Sweden). The number of *Varroa* mites for those colonies was extremely high (192 and 475 individuals, respectively, n = 2) in contrast to other *MEL* colonies (between 7–71 ind., n = 5) and subspecies (*BUCK*: 16–76 ind. (n = 8); *CAR*: 14–61 ind. (n = 8), *LIG*:7–91 ind. (n = 8), min-max values), respectively. Also, the *MEL* colonies where the queen was imported from England showed more *Varroa* mites (159 ind.) at Nordens Ark than the sister colony at Uddevalla (62 ind.). Both colonies with imported *MEL* queens belonged to size class A.

While the subspecies differed significantly in *Varroa* infestation (*F*_*3*,*9*_ *=* 4.14, *p*<0.05), the other factors (Class and Locality) showed no effects (p>0.05) (Fig 4 and S2 Table). In total, nine out of 32 effects were not estimable due to missing observation (NA, Table 3). In the post-analyzed 1-way ANOVA, *MEL* showed high significance for the factor class (F_2,9_ = 4410.1, *p*<0.000), because the highest counts of *Varroa* was mostly found in colonies with a small size (class A) (3 out of 8) (Fig 4), Table 3).

**Table 3:** The number of *Varroa* mites at the two localities.

No of Varroa mites (mean±SD)	Nordens Ark				Uddevalla			
	A	B	C	E	A	B	C	E
*BUCK*	NA (n = 0)	48±4.58 (n = 3)	33±0 (n = 1)	NA (n = 0)	46±0 (n = 1)	22.5±9.19 (n = 2)	76±0 (n = 1)	NA (n = 0)
*CAR*	40±5.66 (n = 2)	21±0 (n = 1)	32±0 (n = 1)	NA (n = 0)	40±0 (n = 1)	51.5±13.44 (n = 2)	14±0 (n = 1)	NA (n = 0)
*LIG*	7±0 (n = 1)	NA (n = 0)	88±4.24 (n = 2)	61±0 (n = 1)	54±0 (n = 1)	53±0 (n = 1)	44±19.80 (n = 2)	NA (n = 0)
*MEL*	317±223.45 (n = 2)	56±0 (n = 1)	7±0 (n = 1)	NA (n = 0)	127±91.92 (n = 2)	71±0 (n = 1)	49±0 (n = 1)	NA (n = 0)

The number of *Varroa* mites in mean±SD for each subspecies (Buckfast hybrid bee of *Apis mellifera* (= *BUCK*), *A*. *m*. *carnica* (= *CAR*), *A*. *m*. *ligustica* (= *LIG*), *A*. *m*. *mellifera* (= *MEL*)) and size class of the colonies (small (= A), medium (= B), large (= C), special case without queen (= E)) of *Apis mellifera* at the two different localities.

### Daily consumption models (DCMs)

Modelling the yield with the adapted metabolic equation (Eq 3) did not perform as well as a simpler linear regression model (Table 1). This indicates that the non-linearity of the metabolic model (Eq 3) is not necessary given the limited temperature range in the winter of 2019–2020. In the linear regression model over temperature the temperature parameter was negative (S3 Table) and the effect was highly significant (S4 Table). Including environmental variables in multiple linear regression models further improved the fit to data. When temperature and light were included in a linear multiple regression model, both the temperature and the light parameters were negative (S5 Table) where the effects were highly significant (S6 Table) and the AIC decreased when compared to the linear regression model with temperature only (Table 1). An intricate picture was presented when looking at the best multiple linear regression DCM, which included the terms wind direction (W), temperature (T), size class (C) and light intensity (L) in the following combinations: W, T:W, T:C, W:L, and T:W:L (S7 and S8 Tables). Many factors were highly significant but the adjusted R^2^ value was low (S7 Table) and all effects were highly significant (S8 Table). Highly significant factors and effects combined with low adjusted R^2^ values provides evidence of relevant factors influencing yield but an inability to predict specific yields.

### Total weight model (TWM)

**Fig 5:**
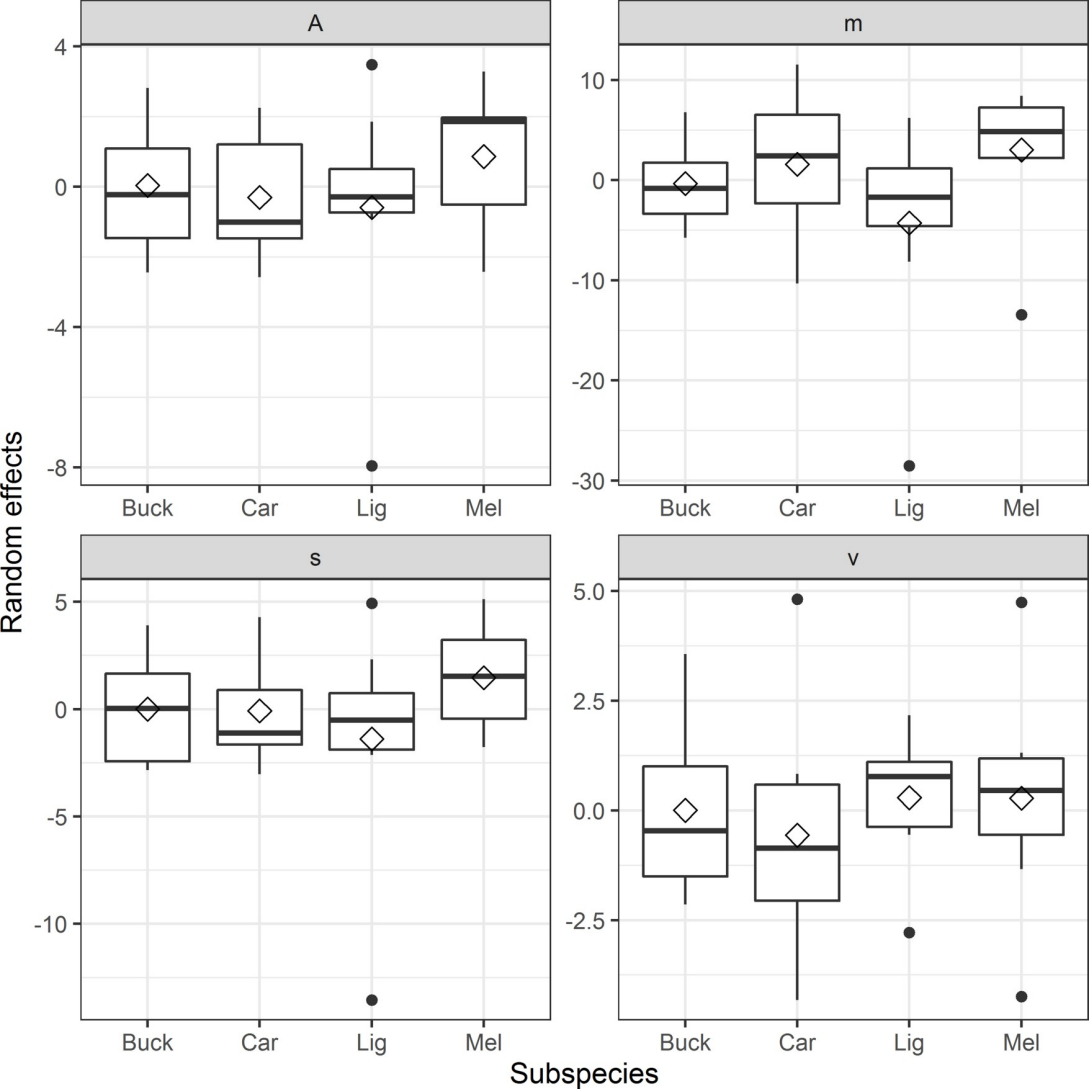
The distributions of the random effects from the total weight model (TWM) with the lowest Akaike information criteria value (AIC). The figure shows the random effects of the lower horizontal asymptote (*A*), the upper horizontal asymptote (*v*), the parameter that describes the day at which the sigmoidal shape is halfway (*m)* and the parameter that governs the slope (*s*). Median values are presented with a black horizontal bar, mean values are shown as a diamond and the interquartile range is shown as a boxplot with outliers as black dots. The subspecies’ abbreviations are the Buckfast hybrid bee of *Apis mellifera* (= *BUCK*), *A*. *m*. *carnica* (= *CAR*), *A*. *m*. *ligustica* (= *LIG*), *A*. *m*. *mellifera* (= *MEL*)).

**Fig 6:**
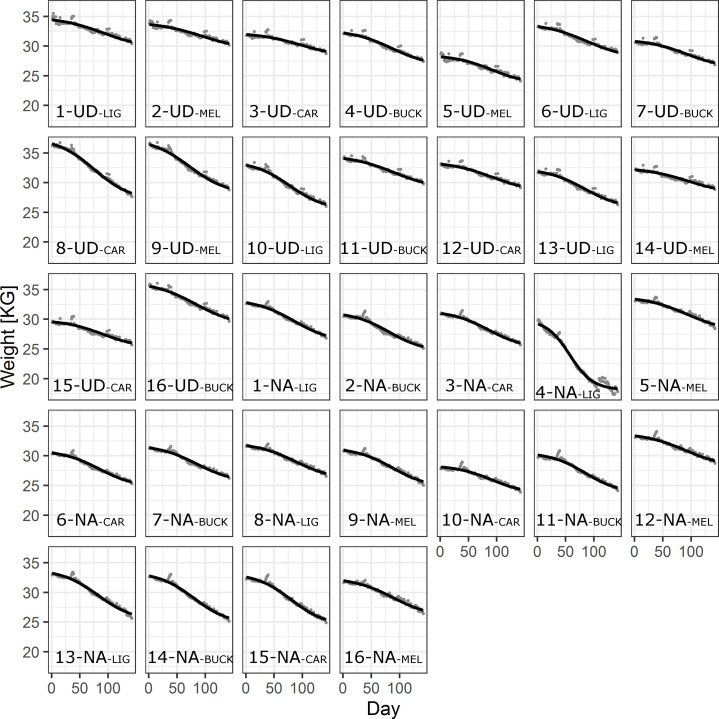
Weight predictions from the best total weight model (TWM) (black lines) over time including data (grey dots) for all beehives. Localities Uddevalla (UD) and Nordens Ark (NA) are identifying the specific beehives (1–16) at each place including description of the subspecies. Beehive 4 at NA was found to be queen-less which likely caused the weight to decrease more than the other colonies.

When modelling weight change over time using the logistic function (Eq 4) the model with the lowest AIC contains an autocorrelation structure of order 1 and a variance structure that allows different standard deviations *per stratum* (Table 1). The fixed effect parameters of this model are *A* = 26.04, *v* = 32.68, *m* = 86.26 and *s* = 33.89 and the distributions of the respective random effects can be seen in Fig 5. Subspecies *MEL* appear to have a higher weight at the lower horizontal asymptote (A) but the variability in the random effects is large and the quartile ranges overlap. The values of the lower and upper horizontal asymptotes (*A* and *v*) are only relevant if we assume that the weight change processes can be extrapolated to the time before and after the time of the experiment and since this is not given, we did not focus on those parameters. Parameter *m* describes the day at which the sigmoidal shape is halfway and *MEL* has the highest median random effect here as well. The variability in the random effects for *m* is small and *MEL*’s interquartile range does not overlap with *BUCK* or *LIG* but does so with *CAR*. Parameter *s* governs the slope of the sigmoidal function and *MEL* once again has the highest median random effects indicating flatter slopes compared to the other subspecies. The resulting predictions capture many of the large-scale patterns in the data well for all beehives (Fig 6).

### Weight assessment models (WAM)

**Fig 7:**
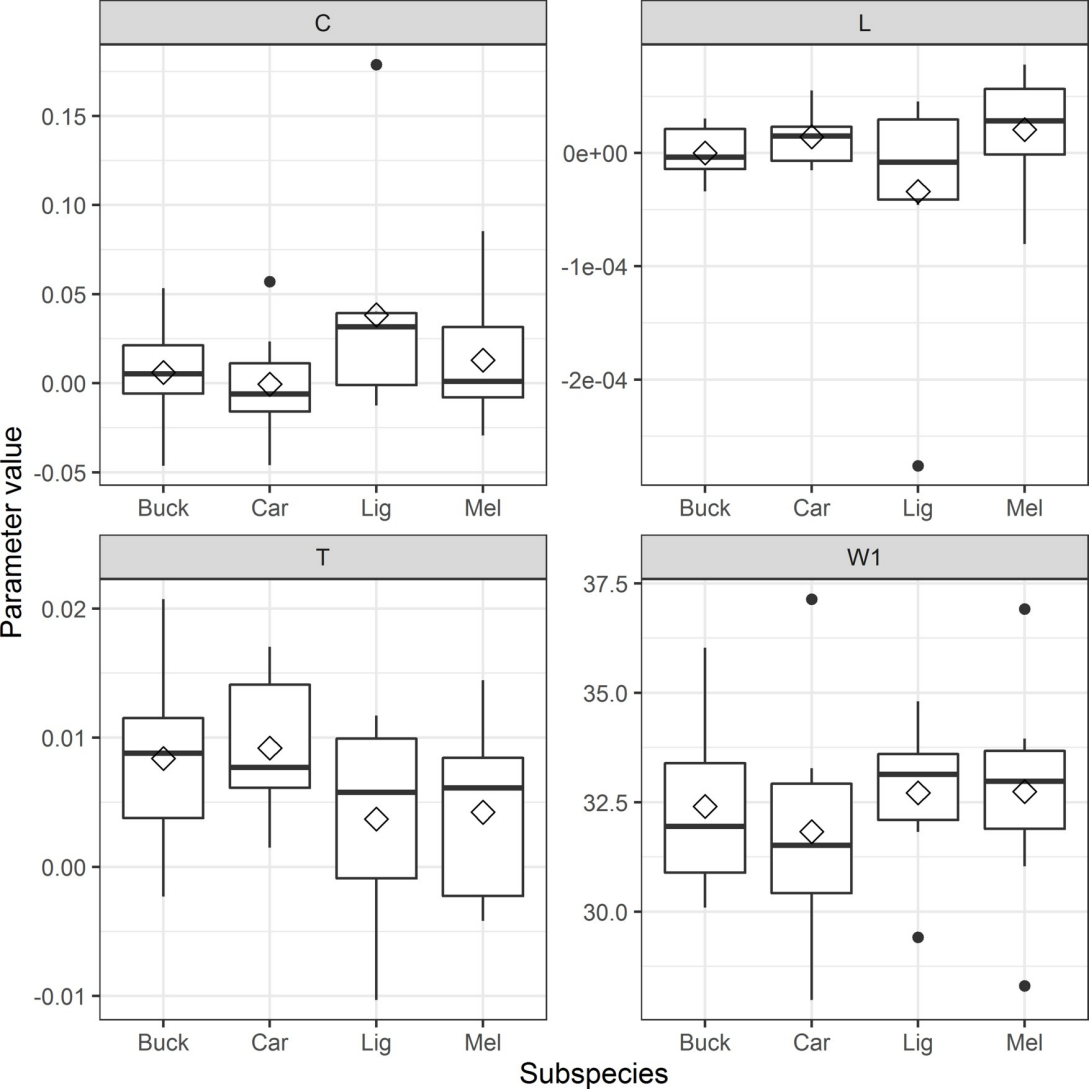
Subspecies specific parameter values of the best weight assessment model (WAM). Median values are presented with a horizontal black bar, mean values are represented with a diamond and the interquartile range is shown as a boxplot with outliers as black dots. The constant consumption rate is represented by parameter *C*, light intensity by parameter *L*, the temperature parameter by *T* and the estimated initial weight of the beehives by *WI*.

When modelling weight over time by simulating consumption one day at a time the model that fits the data best includes the constant, daily, consumption parameter (C), temperature (T) and light intensity (L) (Table 1). The best WAM resulted in average parameter values from all beehives being *W1* = 32.42, *C* = 1.419·10^−2^, *T* = 6.386·10^−3^ and *L* = 2.591·10^−7^. The subspecies-specific median values of the daily consumption parameter *C* in the best WAM varies but *LIG* has the highest median value (Fig 7). Parameter *L* is the parameter determining the impact from light intensity and *MEL* has the highest median value but the interquartile ranges overlap for all species. Since the function of the parameter is negative (see Eq 5) it suggests that *MEL* has the largest weight loss linked to increases in light intensity. The median *L* is negative for *BUCK* and *LIG* but positive for *CAR* and *MEL*. The median temperature effect (*T*) of all subspecies is similar and the interquartile ranges overlap. There are differences between the subspecies in the estimated median initial weight (*W1*) where *MEL* and *LIG* have higher median weights and *BUCK* and *CAR* have lower median initial weights.

**Fig 8:**
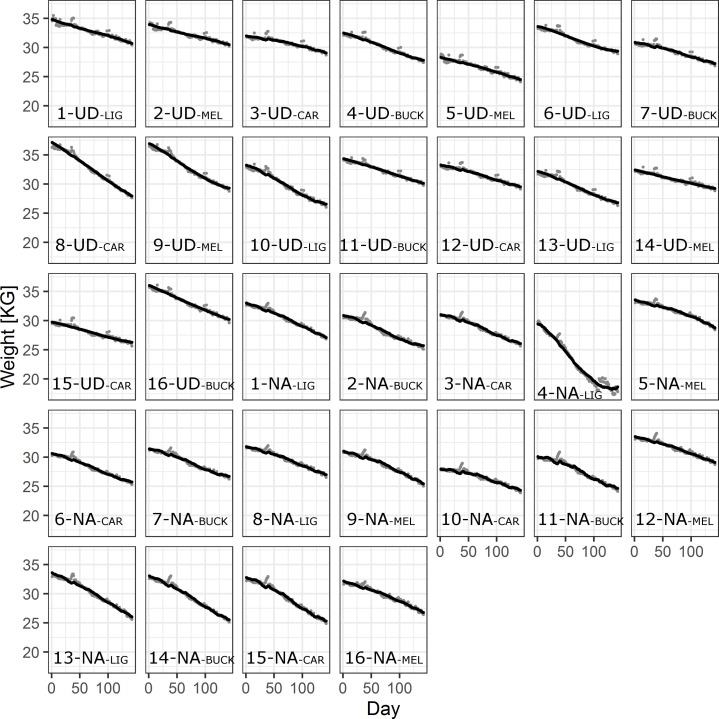
Weight predictions from the best weight assessment model (WAM) (black lines) over time including data (grey dots), for all beehives. Localities Uddevalla (UD) and Nordens Ark (NA) are identifying the specific beehives (1–16) at each place including description of the subspecies.

The resulting predictions manage to capture many of the large-scale patterns in the data well for all beehives (Fig 8).

The residuals over time of the best TWM and the WAM show clear patterns (S1 and S3 Figs) and the residuals over predicted values are not uniformly distributed (S2 and S4 Figs), again limiting the conclusions that can be drawn from the analyses.

The residuals over predicted values of the best TWM and the best WAM both show a group of positive residuals that are not uniformly distributed, lacking corresponding negative residuals (S2 and S4 Figs). These residuals stem from weight increases in the data (grey points in Figs 6 and 8 above the model prediction) which in turn stems from extra weight on the scales, primarily from snow and rain on the top of the beehives. We aimed at avoiding manipulating data and hence we included all such weight gains but one (see Method).

## Discussion

### Weight loss of honey bee colonies

The subspecies *LIG* lost the most weight during the experiment (Fig 3), which partly is due to the one colony being queen-less, which likely led to a less efficient cluster formation for the specific colony and thus less efficiency in heat production and heat maintenance. However, the size class of the colonies had a large impact on the weight loss where large colonies lost more weight (Fig 3). Since *LIG* had the largest colony sizes (Table 2) it follows that *LIG* lost most weight in the experiment. Similarly, *CAR* and *MEL* had the largest number of small colonies (Table 2) and consequently the lowest weight loss. It has been shown that energy consumption per bee in winter clusters decreases in larger clusters, likely due to more efficient heat generation and isolation [44]. In our results, it is clear that smaller colonies consume less resources than the large colonies (Tables 2 and S1). Although the larger colonies can have more efficient *per capita* metabolism there are ultimately more honey bees that need feeding which results in a larger total consumption. Other previous research found that large fall colonies result in large spring colonies [44] and that small colonies in the fall have lower winter survivability [7, 24].

The TWM approach was constructed to deliver a more detailed analysis of the bees’ resource consumption strategies and here the *MEL* subspecies showed the clearest differences. *MEL* appeared to have a slower rate of weight change (a higher *s*) and a later half-day (a higher *m*) compared to the other subspecies, suggesting that *MEL* had a more conservative resource consumption strategy (Fig 5). *CAR* also had a late half-day (a high *m*) but in contrast to *MEL* its random effects showed a higher rate of weight change (a low *s*). The fact that *MEL* has a conservative resource consumption strategy could be indicative of an adaptation to a colder climate, because it is also the subspecies that is native to the Swedish region where winters can be long and where a conservative resource consumption can lower the risk of resource exhaustion. Since our study only included one (exceptionally warm) winter (Fig 2), further winter studies with a larger temperature range are needed to confirm if such an adaptation for this subspecies exists. In general, the consumption rates in our study only differed slightly between the subspecies (*LIG* with the highest and *MEL* with lowest), which is in line with observations in Finland that found a significantly lower winter food consumption for *MEL* in contrast to the other subspecies [45]. In their study, *LIG* and *BUCK* showed the highest winter food consumption.

When comparing overall daily consumption rates (0.039 ± 0.013kg/day) within the experiment and the total weight loss (<9kg) (140 days, average temperature 3.5°C, min-max: -7 to +13°C) with earlier studies, we found that our results better matched weight losses from warmer regions than from regions with colder climates. For example, bees in Iowa/USA also showed an average loss of less than 9 kg (November-April, 150 days) ([46] Russian bees) or from Iowa/Louisiana/Mississippi 6.82 ± 0.34 kg (*A*. *m*. *ligustica*) and 3.93 ± 0.34 kg (Russian bee) (November-April, 147 days) [47]. A study in Japan reported a small weight loss rate of only 0.03kg/day (December—February, *A*. *mellifera*, unknown subspecies) [48]. Another study in Poland (Buckfast and Norwegian *A*. *m*. *m*. x Caucasian bees (= Woznica line)) reported 11.72 ± 3.01 and 9.70 ± 15.03 kg (depending on the subspecies) for uninsulated and 9.72 ± 17.63 and 7.80 ± 25.59 kg for insulated colonies, respectively (November-March, temperature range—7 to +7°C) [49]. Colonies in colder climates in New Haven, Connecticut (*A*. *m*. *ligustica*) and Ithaca, New York (mixed subspecies of *A*. *m*. *ligustica*, *A*. *m*. *caucasica* and *A*. *m*. *carnica*) presented substantially higher weight losses of 23.6±2.8kg (September-mid-April) [12]. We expected a similar high weight loss for Sweden (between 12–20 kg depending on the subspecies), but most likely the main reason for the low daily consumption rate was the abnormally warm winter (Fig 2).

### Varroa infestation

In the last decade, many studies have shown a strong correlation between overwinter losses of honey bee colonies and the intensity of *V*. *destructor* infections (e.g. number of mite infestation) [8, 9, 26, 50]. Also [19], modeled an increased mortality rate per individual, which let the colony collapse earlier, when mites are present. Weight loss due to mite infections was described by [51], but to our knowledge no study has analyzed the effect of *Varroa* intensity on winter weight loss. In our study, we could not find any correlation between weight loss and number of *Varroa* mites. However, we found differences in *Varroa* infestation between the subspecies. In our experiment, *MEL* were more infested than other subspecies. Two colonies with queens originating from the last Swedish *Varroa*-free region (until 2019, Umeå, Northern Sweden) showed the highest infestation rate in our study. During the experiment, it was the first time the queens and their newly produced worker bees were in contact with *V*. *destructor* mites. This would support the theory that uninfected colonies first have to learn and adapt to the stress level of mite infestation. Interestingly, both highly infested *MEL* colonies were small size colonies.

On the island of Gotland, Sweden [52], followed unthreatened colonies with *Varroa* infestations over a decade and could observe a significant decrease of population sizes. Also [50] reported changes in colony sizes when *Varroa* infections were high. Mite infections thus stress the colony and reduce the colony size.

In general, colony size can be an important indicator for colony survival, due to the fact that small bee clusters have a greater ratio of surface area to volume than large ones of the same bee density [44], which means that small colonies must be less effective at preventing heat loss and need to produce more heat. In another study, overwintering success was observed to be highly influenced by size of the colony and weight [9, 24].

### Models and complexity

Interpreting results from ecological non-linear systems, such as honey bee colonies, can be challenging, both due to the complexity of the systems and due to the small number of worked examples in the ecological literature [22]. The three different modelling approaches presented here comes with their own strengths and weaknesses. The DCMs are simple and can provide insights about different environmental variables’ direct effects on daily weight changes. However, to take the cumulative effects of weight loss into account the TWMs and the WAMs were developed. The non-linearity of the TWMs made it difficult to get the model to converge despite the large amount of data, so no other environmental parameters could be included in these models. The WAM approach was therefore developed to be able to simultaneously take environmental parameters and cumulative effects of weight loss into account.

The TWMs are based on Eq 4 which is based on the specific temperature development this specific winter and thus are not easily transferable to other situations. Conversely the WAM approach is flexible enough to be viable for use in other similar studies where further developments, especially with regards to optimization algorithms, will improve the flexibility and thoroughness of the analyses. Since the number of parameters grows quickly in these models a suitable optimization algorithm should be able to deal with large parameter spaces and also preferably be able to include random effects. One suitable software for this would be Template Model Builder (https://CRAN.R-project.org/package=TMB). Despite the limitations in the presented WAMs they, in our opinion, still represent the model approach best suited for further development due to their flexibility.

### Environmental factors

All three model approaches included temperature as a factor influencing the metabolism of the bees, explicitly in the DCMs and the WAMs and implicitly in the TWMs. All models gave the same general result that increasing temperature, within the temperature range of the experiment, increased the honey bees´ weight loss. This indicates that the assumptions behind the metabolic equation (Eq 1) are relevant and hence that it is the improved chemical reactions in the honey bees at higher temperatures that shapes the consumption pattern in the individuals of the clusters at these temperatures. Earlier research of bees’ metabolism under different temperature regimes has shown that in the temperature range of our experiment (-7 to +13°C) the metabolism should decrease as temperature increases and first when temperature is above approximately 10° Celsius metabolism should start to increase with increasing temperatures [4, 17]. The difference in the results could be due to differences in experiment setup, where the previous research maintained the honey bees at specific temperatures for days at a time under laboratory settings, where our colonies were exposed to natural conditions during the whole experiment and thereby taking cumulative effects of resource consumption in the real world into account. It could also be that metabolism is one of several effects (that we have not included in our models) that influence the honey bees’ resource consumption in the winter. However, all our model approaches show, explicitly or implicitly, that when temperature increases, it also increases resource consumption.

When light is included in the DCMs, both temperature and light intensity have negative effects on yield (S5 Table) where including light intensity results in a model fitting the data better (lower AIC), indicating that light intensity is an important factor in understanding honey bee activity and metabolism. This result has similarities to a previous study where temperature and light intensity combined were the strongest predictors of activity at the beehive entrance in the summer [11]. Also [1], pointed out the importance of winter solstice in the annual cycle for bee colonies and if light and temperature are good predictors of activity, both in the summer and during winter, it could indicate that the honey bees utilize these signals as general environmental cues for regulation of activity patterns. In the winter, within the temperature ranges of the experiment, increased temperature increases activity through more efficient chemical processes in the bodies (extending to the clusters) according to the MTE (Eq 1), where the light intensity constitutes a separate environmental signal that can regulate activity. In the summer, increasing temperatures will not have the same physiological effects on metabolism, but when combined with cues of sunlight they could act as indicators of good weather conditions, in order to sync foraging activity with good weather conditions and high nectar productivity in flowers e.g. [53].

The best multiple linear regression DCM included temperature, light intensity, wind direction and size class in different configurations (S7 and S8 Tables) suggesting that wind direction and size class could also be involved in influencing resource consumption, possibly through mechanisms not investigated here or through interactions with mechanisms linked to temperature and light intensity. Wind direction, for instance, could influence the temperature, wind force [49], or the constitution of the air, which can influence the chill effect of the air temperature, or the size of the clusters could influence the efficiency of isolation or heat production e.g. [54].

In the best WAM the subspecies *MEL*’s weight loss effect due to light was the highest, with *CAR* as a close second (Fig 7). It is possible that using light to a larger extent in order to guide resource consumption could be a local adaptation to cold climates, since day length can be a better cue for seasonal change than temperature, especially in areas with dark winters like in Scandinavia. It has been shown that temperature is the predominant factor influencing brood rearing onset in late winter, although it appears to be modulated by photoperiod [13] possibly linking brooding activity to flowering phenology in early spring plants [55]. Plants also use this interaction between light and temperature in the spring to optimize growth and flowering [56], why these observed patterns place emphasis on the co-evolution between the plant and its pollinator in general.

These adaptations to current temperatures and light intensities are at risk of malfunctioning as climate change is projected to alter the temperature while light intensity likely will remain at current levels. If the temperature changes are occurring too fast it would put strain on the overwintering strategies of the bees and their resources which could lead to a miss-match during spring startup, leading to smaller honey bee populations, higher mortalities and, as a consequence, ecosystem services of lesser quality. Additionally, local adaptations to a cold climate could become less successful in a warmer climate which can lead to a shrinking distribution of subspecies with such adaptations. More research on the effects of climate change on honey bees is needed in order for us to be able to mitigate some of these detrimental effects.

## Conclusions

When analyzing winter weight loss in beehives in southwestern Sweden, we found small differences between subspecies but more thorough analyses suggest that *A*. *m*. *mellifera* could have a more conservative resource consumption strategy. Within the temperature range of the experiment (-7 to +13°C) both temperature and light intensity increased weight loss, where the effect of temperature is assumed to stem from more efficient biochemical reactions in the bees at higher temperatures. If both light and temperature influence resource consumption in overwintering honey bee colonies then climate change, with increasing temperatures but similar light conditions, could disrupt the honey bees’ overwintering strategies in Northern climates. Further research is needed if we want to mitigate the negative effects of climate change on honey bee survival.

## Supporting information

S1 File (DS_STORE)

S1 Table Factorial ANOVA of the weight loss. Signification code: p<0.001 = ***.
(XLSX)

S2 Table Factorial ANOVA of the number of Varroa mites. Signification code: p<0.05 = *.
(XLSX)

S3 Table Statistical output of the linear daily consumption model (DCM) over maximum daily temperatures. Residual standard error: 0.2473 on 4542 degrees of freedom. Multiple R-squared: 0.01607, Adjusted R-squared: 0.01586, F-statistic: 74.19 on 1 and 4542 df, p-value: < 2.20E-16. Signification code: p<0.001 = ***.
(XLSX)

S4 Table ANOVA of the linear daily consumption model (DCM) over maximum daily temperature. Signification code: p<0.001 = ***.
(XLSX)

S5 Table Statistical output of the linear daily consumption model (DCM) over maximum daily temperatures and light intensity. Residual standard error: 0.246 on 4541 degrees of freedom. Multiple R-squared: 0.02647, Adjusted R-squared: 0.02604, F-statistic: 61.74 on 2 and 4541 df, p-value: < 2.20E-16. Signification code: p<0.001 = ***.
(XLSX)

S6 Table ANOVA of the linear daily consumption model (DCM) over maximum daily temperature and light intensity. Signification code: p<0.001 = ***.
(XLSX)

S7 Table Statistical output of the best daily consumption model (DCM) multiple linear model. *W* represents the wind direction with the number describing the degrees which are divided into eight steps (0, 44, 90, 134, 180, 224, 270, 314). Size class is denoted CB = size class B, CC = size class C and CE = size class E. Interaction between terms is denoted with “:”. Residual standard error: 0.2359 on 4509 degrees of freedom. Multiple R-squared: 0.1112, Adjusted R-squared: 0.1045, F-statistic: 16.59 on 34 and 4509 DF, p-value: < 2.2e-16. Signification codes: p<0.001 = ***, p<0.01 = **, p<0.05 = *.
(XLSX)

S8 Table ANOVA of the best multiple linear daily consumption model (DCM). *W* represents the wind direction, T is temperature and C is size class. Interaction between terms is denoted with “:”. Signification codes: p<0.001 = ***, p<0.01 = **.
(XLSX)

S1 Fig Residuals of the best total weight model (TWM) over time. (TIFF)

S2 Fig Residuals of the best total weight model (TWM) over predicted values. (TIFF)

S3 Fig Residuals of the best weight assessment model (WAM) over time. (TIFF)

S4 Fig Residuals of the best weight assessment model (WAM) over time. (TIFF)
