# Supplementary figures and images for: Winter weight loss of different subspecies of honey bee Apis mellifera colonies (Linnaeus, 1758) in southwestern Sweden
Source: PLoS One. 2021 Oct 14;16(10):e0258398. doi: 10.1371/journal.pone.0258398 (PMC8516218; doi:10.1371/journal.pone.0258398)

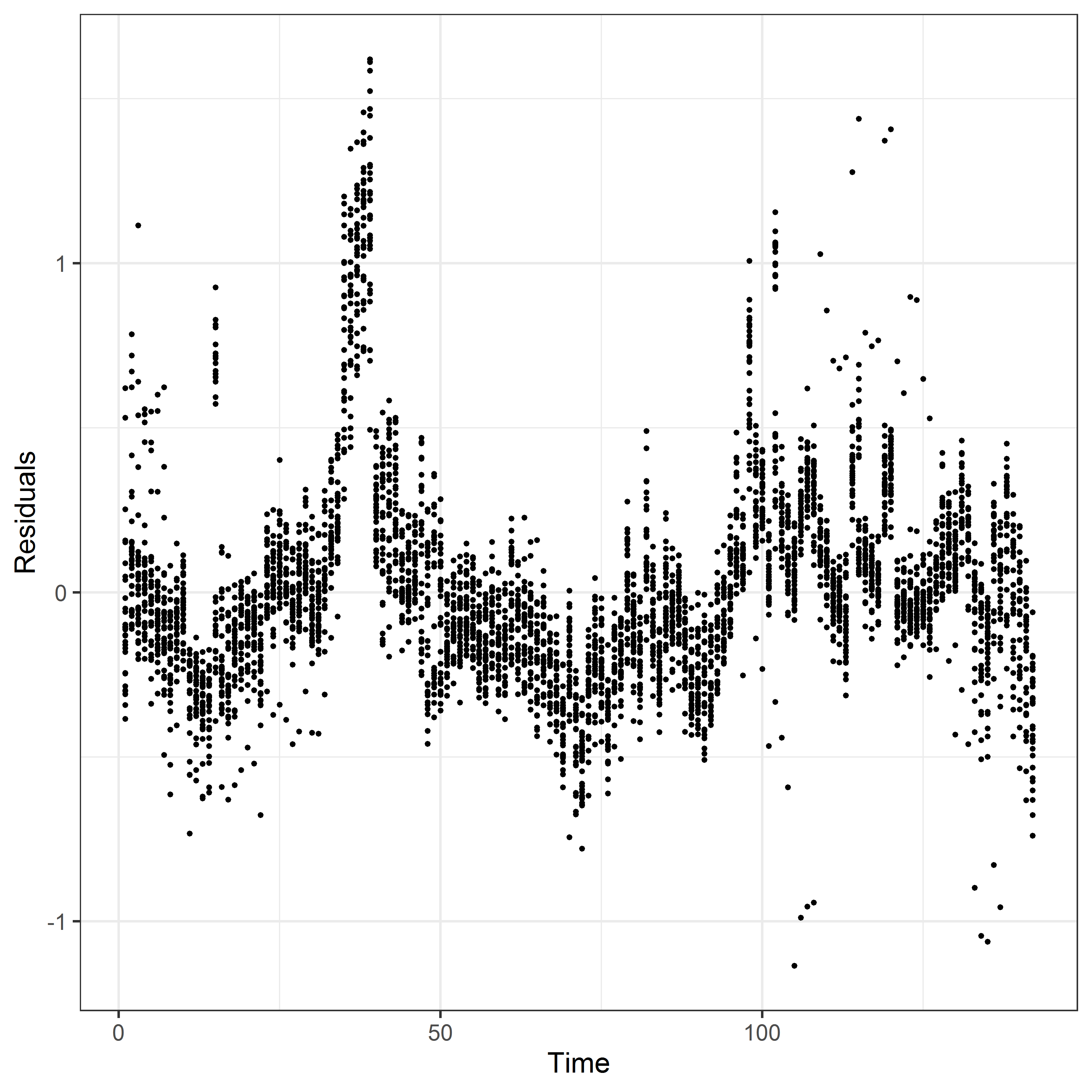

Supplement: S1 Fig — (TIFF) [file pone.0258398.s010.tiff]

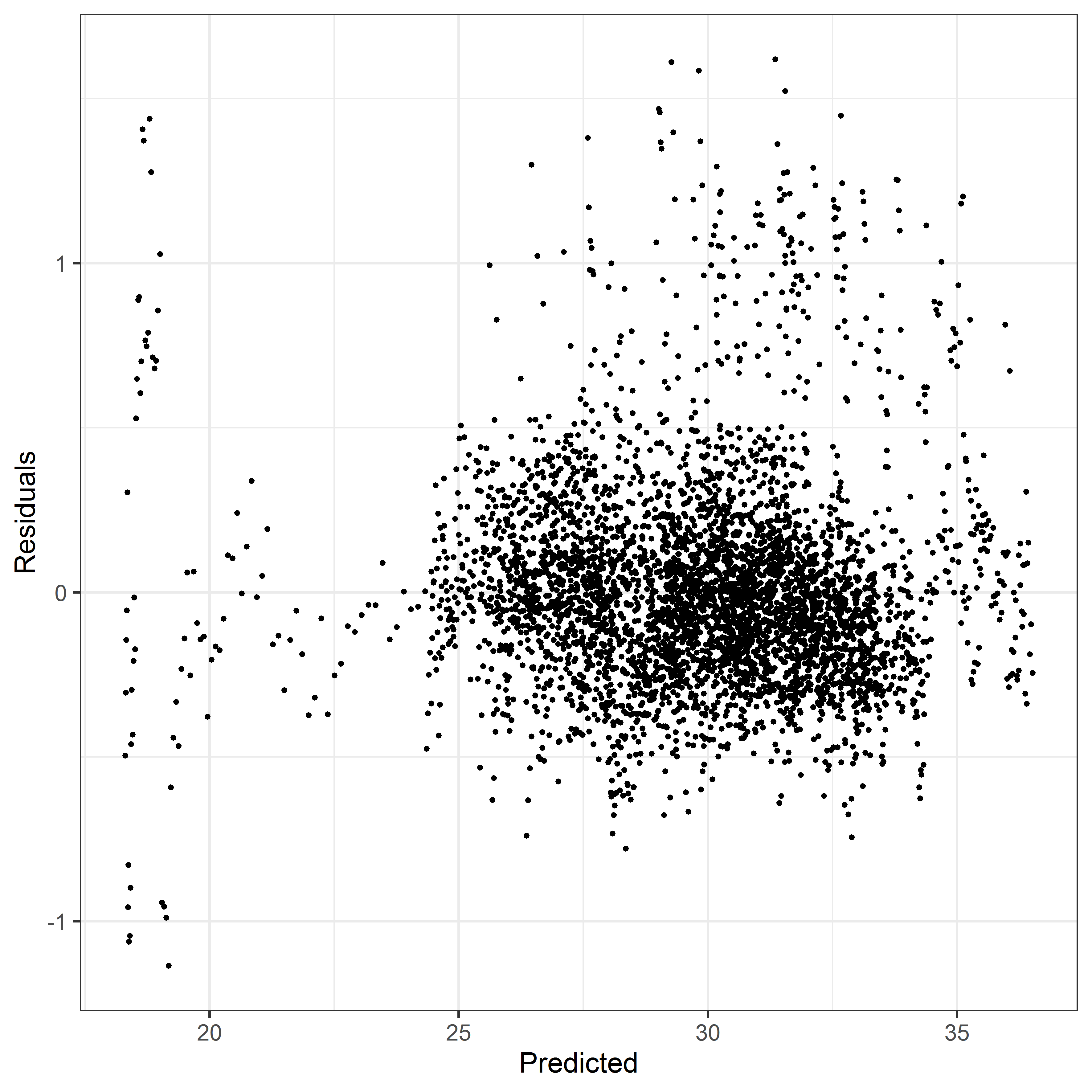

Supplement: S2 Fig — (TIFF) [file pone.0258398.s011.tiff]

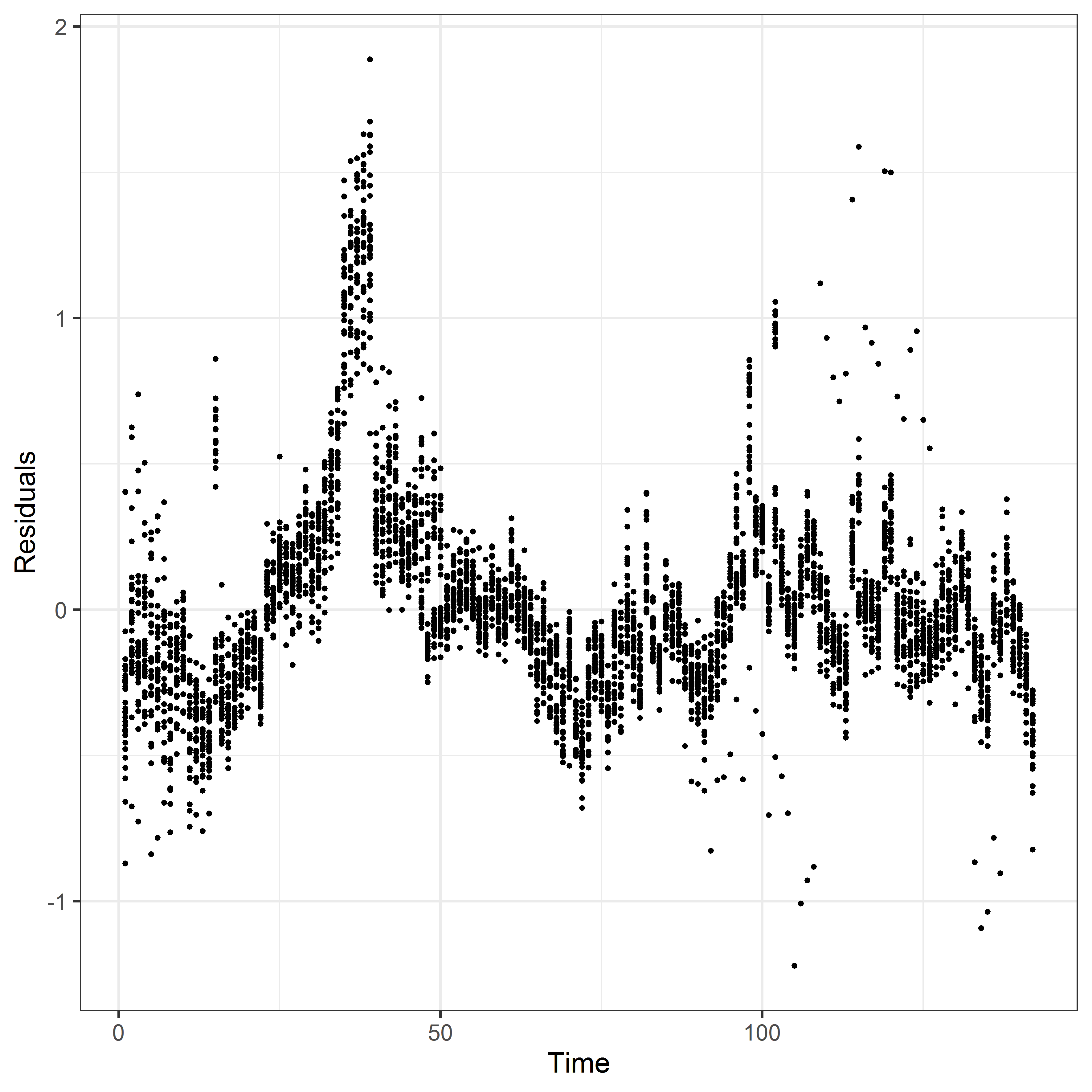

Supplement: S3 Fig — (TIFF) [file pone.0258398.s012.tiff]

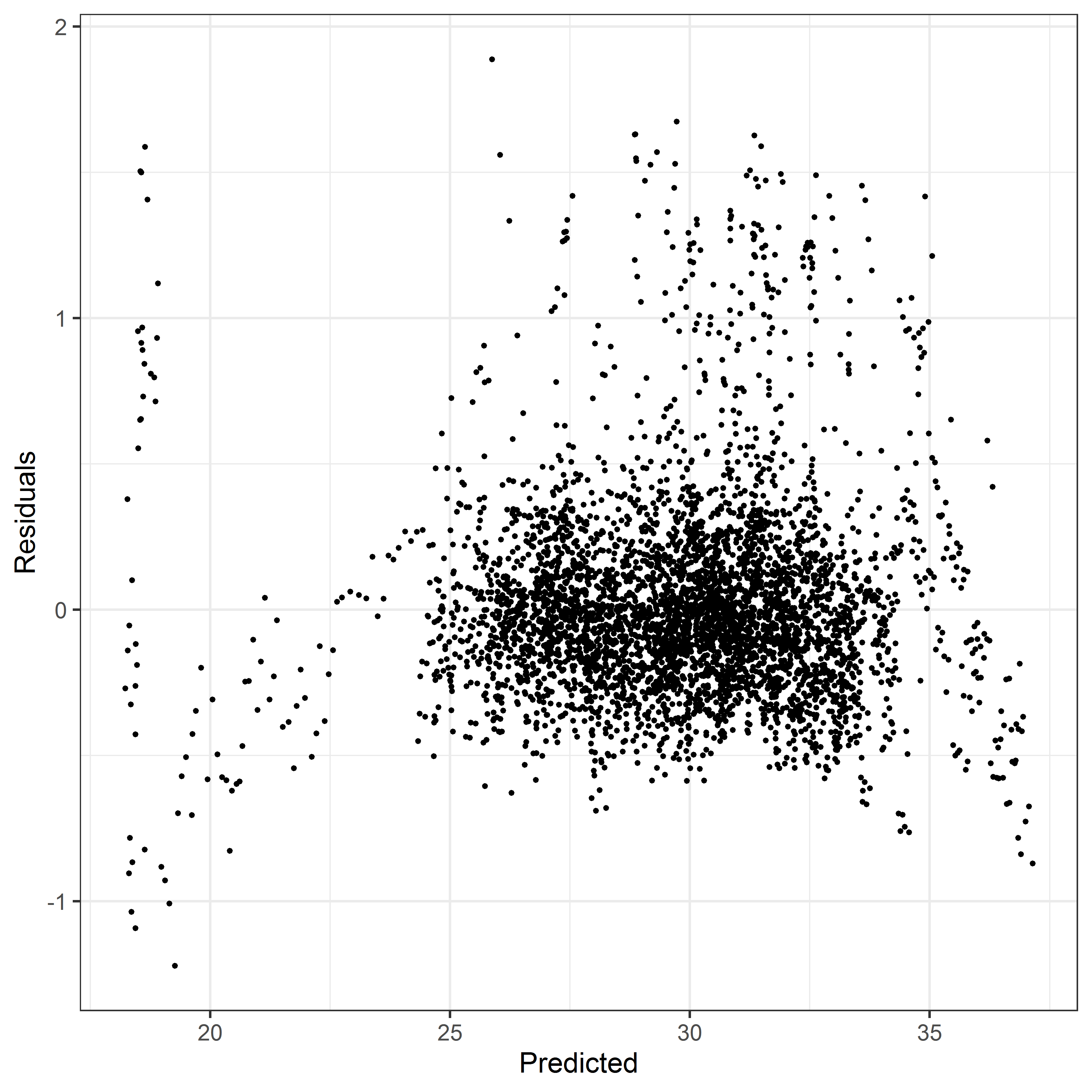

Supplement: S4 Fig — (TIFF) [file pone.0258398.s013.tiff]
